# Valorization of Defatted Cherry Seed Residues from Liquor Processing by Matrix Solid-Phase Dispersion Extraction: A Sustainable Strategy for Production of Phenolic-Rich Extracts with Antioxidant Potential

**DOI:** 10.3390/antiox12122041

**Published:** 2023-11-24

**Authors:** Sandra Rodríguez-Blázquez, Lorena Fernández-Ávila, Esther Gómez-Mejía, Noelia Rosales-Conrado, María Eugenia León-González, Rubén Miranda

**Affiliations:** 1 Department of Analytical Chemistry, Faculty of Chemistry, Complutense University of Madrid, Avda. Complutense s/n, 28040 Madrid, Spain; sandro08@ucm.es (S.R.-B.); lorenalf@ucm.es (L.F.-Á.); egomez03@ucm.es (E.G.-M.); leongon@ucm.es (M.E.L.-G.); 2 Department of Chemical Engineering and Materials, Faculty of Chemistry, Complutense University of Madrid, Avda. Complutense s/n, 28040 Madrid, Spain; rmiranda@ucm.es

**Keywords:** by-products, *Prunus avium* L., defatted cherry seeds, liquor processing, polyphenols, matrix solid-phase dispersion, HPLC-ESI-MS/MS, antioxidant properties

## Abstract

The integrated valorization of food chain waste is one of the most promising alternatives in the transition to a sustainable bioeconomy. Thus, an efficient solid-phase matrix dispersion extraction method, using experimental factorial design and response surface methodology, has been developed and optimized for the recovery of polyphenols from defatted cherry seeds obtained after cherry liquor manufacture and subsequent fatty acid extraction, evaluating the effect of each processing step on the composition and phenolic content of sweet cherry residues. The phenolic extracts before fermentation showed the highest content of total polyphenols (TPC) and flavonoids (TFC) (3 ± 1 mg QE·g^−1^ and 1.37 ± 0.08 mg GAE·g^−1^, respectively), while the highest antioxidant capacity was obtained in the defatted seed extracts after both fermentation and distillation. In addition, high-performance liquid chromatography coupled to a quadrupole time-of-flight mass spectrometer (HPLC-ESI-QTOF-MS) was used to determine the phenolic profile. Dihydroxybenzoic acid, neochlorogenic acid, caffeic acid, and quercetin were the main phenolics found, showing differences in concentration between the stages of liquor production. The results underline the prospective of cherry by-products for obtaining phenol-rich bioactive extracts for possible use in different industrial sectors, offering a feasible solution for the cascade valorization of cherry agri-food waste.

## 1. Introduction

*Prunus avium* L. is a deciduous tree belonging to the family Rosaceae, which produces one of the most popular and appreciated red temperate fruits among consumers, the sweet cherry [1]. Its bright red color, flavor, aroma, desirable organoleptic properties, and high nutritional quality and bioactive properties have led to a significant increase in consumer demand [2,3]. A very common practice in cherries is the production of cherry liquor and distilled spirits (such as Kircshwasser, ceresznye pálinka, or brandy) from out-of-specification fruit that cannot be marketed. The resultant liquor presents a characteristic pleasant aroma and taste [4]. Cherry liquors are made by maceration processes, i.e., the fruit is mixed in its own spirit (37.5% up to 70% (*v*/*v*) ethanol), which is obtained by fermentation and subsequent distillation from cherry must (freshly squeezed cherry juice containing the skins, stones, and stems), obtaining the liquor with an alcoholic concentration of 15–17° (*v*/*v*) [5]. During this practice, the stones are kept intact, being one of the main residues generated in the production of liquor [6]. Especially in The Jerte Valley (Extremadura, Spain), where more than 60 wt% of Spanish sweet cherry is produced (22,000 tons per year), it is estimated that approximately 143 tons per year of sweet cherry stone waste is generated during the production of Kirsch (cherry brandy) [6,7].

Cherry stones, a by-product of liquor production, contain a high added-value component known as seed or kernel, which accounts for 15–30 wt% of the entire stone [4,5,8]. Sweet cherry seeds contain important nutritional components and high levels of bioactive compounds such as polyphenols, flavonoids, fatty acids, tocopherols, glucose, fructose, vitamin C, carotenoids, and anthocyanins with various health benefits [9,10]. One of the possible and common applications of this agri-food waste is the extraction of oils rich in unsaturated fatty acids. However, during the process of obtaining oil, a solid residue is generated that could contain substances of interest, such as polyphenols. The extraction of the polyphenol-enriched residue in this hydrophilic step could be of interest in accordance with the circular economy concept [11]. However, there is a lack of information on this cascade valorization process of sweet cherry seeds.

As previously reported, phenolic compounds, such as phenolic acids (hydroxycinnamic derivates) and flavonoids (anthocyanins, flavan-3-ols, and flavanols), are one of the most interesting components of sweet cherries. Sweet cherries contain more than 200 mg of polyphenols/100 g of fruit [12]. One of their main phenolic compound groups is anthocyanins. Besides anthocyanins, some phenolic acids identified in this fruit are neochlorogenic acid, chlorogenic acid, *p*-coumaric acid, and caffeic acid [9,13,14,15]. Also, different types of flavonoids are found in this variety of cherry, such as catechin, epicatechin, quercetin, quercetin-3-glucoside, rutin, and kaempferol derivative [9,12,13,14,16,17]. The presence of these phytochemicals is linked to the prevention of chronic and degenerative diseases [9]. Polyphenol intake has been shown to be inversely related to the risk of cardiovascular disease and all-cause mortality in subjects at high cardiovascular risk and cancer [18]. In this respect, sweet cherry seeds exhibit important bioactive activities such as anti-aging, antimicrobial, anti-tumor, or anti-inflammatory, and a consequence of preventing cell oxidative damage [18,19]. Alfonso et al. [9] studied the extracts of cherry seeds and established that their antioxidant activities with radical scavenging assays using 2,2′-azino-bis-3-ethylbenzothiazoline-6-sulfonic acid (ABTS), 2,2-diphenyl-1-picrylhydrazyl (DPPH) radical, and the ferric reducing activity power (FRAP). The antioxidant activities were correlated with the phenolic content and exhibited high antimicrobial activity against important human insolates (*Staphylococcus aureus* MJS241 and *Enterococcus faecalis* MJS257). Furthermore, Concepción et al. [20], Antognoni et al. [21], and Agulló-Cazarra et al. [22] indicated that cherry seeds present interesting antioxidant properties, such as anti-hypertensive properties, photoprotective cellular activities, or anti-aging capacities, or the property of counteract cellular oxidative stress. Therefore, great interest is shown currently in recovering this type of natural compound from agri-food waste [8]. Notwithstanding, there is no literature information on the phenolic profile and bioactive activities of sweet cherry seed residues generated during the production of cherry liquor. Nevertheless, changes in the phenolic content of seeds and their antioxidant activity could be affected during fermentation and distillation processes. The effect of fermentation was studied in cocoa beans by Nazaruddin et al. [23], who reported that polyphenols diffuse and undergo oxidation to condensed high molecular, mostly insoluble tannins during fermentation. Moreover, the content of catechin and epicatechin was reduced during the fermentation process [23]. Regarding distillation, Cisneros-Yupanqui et al. [24] reported that the total polyphenol content in grape pomace decreased after the distillation process due to the high temperatures employed.

Hence, in this context, the extraction and subsequent characterization of polyphenols needs to be developed. Nowadays, there is an increasing urge for faster and more environmentally friendly methods, including ultrasonic-assisted extraction (USE), microwave-assisted extraction (MAE), and matrix solid-phase extraction (MSPD) [16,18,19]. In this line, MSPD is a fast, flexible, versatile, and simple technique, which consists of mixing and grinding the sample with a suitable solid support for subsequent desorption of the analytes with a small amount of elution solvent [25]. Despite the outstanding advantages of this extraction technique, it has so far not been successfully applied to sweet cherry defatted seed residues. On the other hand, experimental design and response surface methodologies are necessary for the optimization of the extraction method [25,26].

Hence, the aim of the present work was to develop a sustainable polyphenol MSPD extraction method from the defatted seed residues of sweet cherry generated during the production of cherry liquor, making it possible to evaluate the effect of liquor processing on phenolic recovery. A factorial experimental design with replicates at the center point and response surface analysis methodology was used to optimize the MSPD extraction conditions. The phenolic characterization of the resultant extracts at the different steps of cherry liquor processing was accomplished by HPLC-ESI-QTOF-MS as well as by spectrophotometric methods, which allowed studying the differences in the phenolic composition extracts and to evaluate the influence of each processing step. In addition, the antioxidant potential of the defatted sweet cherry seed extracts was investigated to explore the possible cascade valorization of the liquor biowaste to obtain high-value-added products.

## 2. Materials and Methods

### 2.1. Deffated Cherry Seeds (Prunus avium L.) before Fermentation, after Fermentation, and after Both Fermentation and Distillation

The defatted cherry seeds of the variety *Prunus avium* L. before fermentation, after fermentation, and after both fermentation and distillation were the bioresidue investigated in the present work.

Cherry stones of the variety “*Prunus avium* L.” before fermentation, after fermentation, and after both fermentation and distillation in cherry liquor processing were provided by The Jerte Valley Cooperatives Group (Cáceres, Spain, 2022 campaign). The cherries used to produce cherry liquor were crushed with rubber rollers without breaking the stone. The resulting stones, together with the pulp, were subjected to a stage of biological fermentation of the mash at a controlled temperature (18–20 °C) for 25–30 days. Subsequently, a conventional batch distillation process was carried out using indirect heat in batch-loaded stills.

The procedure described by Rodríguez-Blázquez et al. [3] was followed for pretreatment of the cherry pits. Cherry pits, derived from the three processes, were air-dried at 40 °C (Digitheat oven, J.P Selecta^®^, Abrera, Barcelona, Spain) for 24 h and separated manually, using a hammer, into shell and seed. The seeds were then crushed in an ultracentrifuge crusher (Retsh™ ZM200, Haan, Alemania) and sieved with a stainless-steel sieve to particle sizes below 1 mm. Then, the initial moisture content of cherry seeds was determined according to the standard procedure AOAC 925.10 [27], with slight modifications. Approximately 2 g of seed sample was weighed on a dried crucible and introduced in an oven at 105 °C for 2 h and 30 min until a constant weight was obtained. After cooling, the crucible was weighed again, and the free water content was calculated as sample weight loss and expressed as a percentage in weight (mean ± standard deviation, *n* = 3). Finally, the cherry seeds were defatted according to the Soxhlet extraction procedure (AOAC 960.39) [28]. The tests were carried out in duplicate, and the defatted seeds content was determined and expressed as mean in weight ± standard deviation percentage. The defatted seeds were stored in clear plastic zip-lock bags until analysis.

### 2.2. Reagents, Solvents, and Polyphenol Standards

Analytical-grade reagents and purified water from a Milli-Q system (Merck, Madrid, Spain) were used. *n*-Hexane (96%), ethanol absolute (EtOH), acetonitrile (ACN), methanol (MeOH), and formic acid (FA) of MS quality were provided by Scharlab (Barcelona, Spain). Dimethyl sulfoxide (DMSO, ≥99.9%), 2N Folin–Ciocalteu reagent, 2,2-diphenyl-1-picrylhydrazyl (DPPH), trichloroacetic acid, and thiobarbituric acid were all supplied by Sigma-Aldrich (St. Louis, MO, USA). Aluminum chloride 6-hydrate, L-ascorbic acid, sulphuric acid (95–98%), sodium carbonate anhydrous, sodium hydroxide, and sodium nitrite were obtained from Panreac (Barcelona, Spain).

Phenolic standards gallic acid monohydrate (≥98.0%), dihydroxybenzoic acid (≥97.0%), chlorogenic acid (≥95.0%), catechin (≥98.0%), caffeic acid (≥98.0%), *p*-coumaric acid (≥98.0%), epicatechin (≥98.0%), *trans*-ferulic acid (98%), rutin trihydrate (≥95.0%), myricetin (≥96.0%), resveratrol (≥99.0%), quercetin (≥95.0%), kaempferol (≥97.0%), and naringin (≥95.0%) were obtained from Sigma-Aldrich (St. Louis, MO, USA). Hesperidin (≥98.0%) was provided by European Pharmacopoeia. Phenolic stock solutions (200 mg·L^−1^) were prepared in MeOH, ethanol–water mixture 80:20 (*v*/*v*) (quercetin), or 5% (*v*/*v*) DMSO aqueous solution (hesperidin). They were stored in the dark at 4 °C or at −80 °C (myricetin, hesperidin, *trans*-ferulic, and caffeic acid) for up to one month. Fresh working standard solutions were prepared daily by diluting stock solutions as needed.

### 2.3. Extraction of Phenolic Compounds from Residual Cherry Seeds

With the aim of determining the most influential factors in the recovery of polyphenols from defatted cherry seed residues, a series of preliminary tests were carried out using the sweet cherry seeds prior to fermentation as a model. The extraction factors studied were the nature of the dispersant diatomaceous earth (Restek Corporation) and silica (Fluka), the sample–dispersant ratio (1/1 and 1/4 (*w*/*w*)), and the stirring mode: ultrasonic bath (P-Selecta) and vortex (WX, VELP Scientifica). In contrast, the responses considered were total flavonoid content (TFC) and DPPH scavenging antioxidant activity. Thereafter, the input factors and responses were subjected to a multifactorial analysis of variance (ANOVA) to determine the significance of the factors in the extraction of phenols form the residual cherry seed.

Then, in accordance with Gómez-Mejía et al. [18], a two-level, three-variable factorial design was carried out with three replicates at the central point. The ethanol content of the extraction solvent (20–80%, *v*/*v*), stirring time interval (5–10 min), and the sample-to-silica ratio (1/1–1/5 (*w*/*w*)) were chosen as experimental factors. The upper and lower limits of the evaluated factors were established considering the results obtained in the preliminary tests and were normalized by assigning the values −1, 0, and 1. The experimental responses were the DPPH antiradical activity, TPC, and TFC. Consequently, a total of 11 experiments (2^3^ = 8 experiments with three replicates at the central point) were performed, comprising 11 runs of the full factorial experimental design (Table 1). This factorial model has been proposed as a screening method to determine the starting conditions for a subsequent scaling process with a reduced number of experiments [29]. In addition, the replicates at the central point allow determining the reproducibility of the model [29,30]. The input variables and outcomes were then subjected to ANOVA and linear regression analysis to evaluate the relevance of the used model and its repeatability. Finally, optimization was carried out to establish the factors that maximize the aforementioned responses, i.e., promote the total indexes and minimize the concentration of sample required to inhibit 50% of the initial DPPH concentration (IC_50_ value) of the antioxidant activity.

The analysis of the planned experimental factorial design (Table 1) made it possible to determine the most important factors influencing the selected experimental responses, as well as their interaction effects.

Total polyphenol content, total flavonoid content, and DPPH scavenging antioxidant capacity (IC_50_) were correlated with the experimental factors using standardized polynomial equations (Equation (1)).

(1) $$Y=\beta_{0}+\beta_{1}x_{1}+\beta_{2}x_{2}+\beta_{3}x_{3}+\beta_{12}x_{1}x_{2}+\beta_{13}x_{1}x_{3}+\beta_{23}x_{2}x_{3}$$

where Y is the predicted response, $\beta_{0}$ is the intercept, $\beta_{1}$, $\beta_{2},$ and $\beta_{3}$ are the linear coefficients of $x_{1}$ (sample–dispersant ratio (*w*/*w*)), $x_{2}$ (ethanol–water ratio (*v*/*v*)), and $x_{3}$ (vortex stirring time).

### 2.4. MSPD Optimized Extraction Method

The optimized MSPD extraction process consisted of blending 0.1000 g of defatted sweet cherry seeds and 0.1000 g of silica in a porcelain mortar for 2 min. The solid mixture was then transferred to a 5 mL plastic tube with 3 mL of the EtOH-H_2_O 80:20 (*v*/*v*) and stirred for 10 min at 402.48× *g*, using a vortex. Lastly, a clear supernatant was obtained after centrifugation for 30 min at 1528× *g* (centrifuge 5804, Eppendorf). Samples were prepared in triplicate.

### 2.5. Total Phenolic Content

The *Folin–Ciocalteu* method was used to determine the total phenolic content of cherry seed extracts [31]. The phenolic extracts (750 µL) were combined with 70 µL of the Folin–Ciocalteu reagent, 60 µL of 7.5% (*w*/*v*) Na_2_CO_3_, and Milli-Q water to a final volume of 10 mL. For external calibration, gallic acid was employed as the standard (0–40 µM, *n* = 5). The absorbance of the solutions was measured at 720 nm (Thermo Scientific Multiskan spectrophotometer, Agilent Technologies), and the results were expressed as mg of gallic acid equivalent per g of dry sample (mg GAE·g^−1^). The assay was performed in triplicate.

### 2.6. Total Flavonoid Content

The aluminum complexation colorimetry test was used to assess the total flavonoid content [18]. Sample extracts (750 µL) were added to 2 mL of Milli-Q water, 150 mL of 5% (*w*/*v*) NaNO_2,_ and 150 mL of 10% (*w*/*v*) AlCl_3_. After 15 min of storage and two 5 min incubation periods, 1 mL of 1 M NaOH was added to stop the reaction solution, which was then diluted to 10 mL with Milli-Q water. Using a UV-Vis spectrophotometer, the absorbance was measured at 415 nm, and the results were represented as mg of quercetin equivalent per g of dry defatted seed (mg QE·g^−1^), using quercetin as standard (0–45 µM, *n* = 6). Samples were analyzed three times.

### 2.7. Antioxidant Activity

The antioxidant activity of defatted sweet cherry seed phenolic extracts was evaluated based on the ability of the extracts to scavenge the DPPH free radical and to inhibit the lipid peroxidation, as previously described by Gómez-Mejía et al. [18], with minor modifications.

As for the DPPH method, working solutions were prepared in a 96-well microplate by combining 100 µL of 0.28 mM DPPH (MeOH) with 10–100 µL of samples aliquots to a final volume of 200 µL, using EtOH-H_2_O 80:20 (*v*/*v*) as solvent. Additionally, a blind control (sweet cherry seeds combined with MEOH pure) and a DPPH control were prepared. Trolox was used as a reference compound (positive control). The absorbance was measured at 515 nm following 60 min incubation at room temperature in the dark. Finally, the IC_50_ value was determined after three independent tests and expressed as mg of extract per g of dry seed.

The thiobarbituric acid reactive substance (TBARS) colorimetric assay consisting of incubating 100 μL porcine brain homogenates (20 mM Tris-HCl buffer, pH 7.4, 1:2 (*w*/*v*)) with extract working solutions (200 μL, 4.0–0.125 mg·mL^−1^), FeSO_4_ (100 μL, 1.52 mg·L^−1^) and 100 μL of ascorbic acid (17.6 mg·L^−1^). Additionally, a blank, a TBARS control solution (water), and a reference compound (trolox) with a known antioxidant capacity were prepared. After 1 h incubation at 37.5 °C, 500 μL of trichloroacetic acid (28% (*w*/*v*)) and 380 μL of thiobarbituric acid (2% (*w*/*v*)) were added, and the mixture was maintained at 80 °C for 20 min. Finally, the absorbance of the supernatant was measured at 532 nm. Each assay was performed in triplicate.

### 2.8. Chromatographic Analysis of Phenolic Extracts by HPLC-ESI-QTOF-MS

The individual determination of polyphenols from the phenolic extract of defatted sweet cherry seeds at the different steps of liquor processing, obtained under optimal MSPD extraction conditions, was conducted by high-performance liquid chromatography coupled to a quadrupole time-of-flight mass spectrometer (HPLC-ESI-QTOF-MS). The analysis was performed on an Agilent liquid chromatography system (Mod. 1200) comprised of a quaternary pump (G1311A), a coupled degasser (G1322A), an automatic injector with thermostat (G1367B), a column module with thermostat (G1316A) and a QTOF mass spectrometer (Agilent G6530A) with electrospray ionization (ESI) source at atmospheric pressure and JetStream technology, operating in negative mode and scanning mode (SCAN) in the *m*/*z* 100–1000 range, using a capillary voltage of 4 kV and a pressure of 45 psi. Data processing was performed with Masshunter Data Acquisition B.05.00, Masshunter Qualitative Analysis B.07.00, and Massprofinder Professional B.08.00. Nitrogen was used as the fogging and drying gas (10.0 L·min^−1^, 325 °C).

The HPLC-QTOF separation was carried out using a Synergi™ C18 Fusion-RP 80 Å analytical column (150 mm × 3 mm I.D., 4 μm, Phenomenex, Torrance, CA, USA), kept at 30 °C, the flow rate was set at 0.50 mL·min^−1^ and the injection volume was fixed at 20 μL, following the method previously described by Gómez-Mejía et al. [32]. Briefly, a mobile phase gradient based on a combination of 0.1% (*v*/*v*) formic acid (FA) in distilled water (solvent A) and 0.1% (*v*/*v*) FA in acetonitrile (ACN) (solvent B) was employed as follows: 10% of the solvent B was kept for 0.1 min, then increased linearly to 35% in 30 min, and reached 70% in 5 min. This state was held for 2 min, after which a final linear rise to 90% B was obtained in 3 min and sustained for 5 min. Eventually, the gradient was re-equilibrated. The identification of phenolic compounds was based on the high-resolution mass data collected from commercial standards and databases (FooDB and Mass Bank). For quantification purposes, 5-level external calibration curves were obtained for phenolic standards. Whenever standards were not available, a semi-quantification was performed using the most similar standard available. Finally, the results were expressed in mg per gram of dry sample.

### 2.9. Statistical Analysis

Data were statistically analyzed by one-way analysis of variance (ANOVA), multifactorial ANOVA, the least significant difference (LSD) multiple comparison test, and principal component analysis (PCA) using the software package Statgraphics 19 (Statgraphics Technologies. Inc., Rockville, MD, USA).

## 3. Results

### 3.1. Moisture Content of the Defatted Sweet Cherry Seeds

The moisture content in the defatted seed samples was determined following the AOAC Official Method 925.10 [27] indicated in Section 2.1. The percent moisture content determined for the cherry seed residues analyzed were (3.6 ± 0.1%, w) for the seed before fermentation and (3.3 ± 0.2%, w) and (2.4 ± 0.4%, w) for the seed after fermentation and after both fermentation and distillation, respectively.

### 3.2. Defatted Seed Yield of Sweet Cherry Seeds

The defatted seed content, resulting from the Soxhlet extraction procedure [28] with *n*-hexane as a solvent, was determined in the cherry seed residues from before fermentation, after fermentation, and after both fermentation and distillation processes. The percentages in weight of defatted seed extraction were (61 ± 3%, w) for the seed from before fermentation, (54 ± 3%, w) for the seed after fermentation, and (46.3 ± 0.4%, w) for the seed after fermentation and distillation. All results showed that during Soxhlet extraction, a large amount of seed residue is generated that could be valorized to obtain high added-value compounds, such as polyphenols [33].

### 3.3. MSPD Extraction Method Optimization

Defatted sweet cherry seed (*Prunus avium* L.) from before the fermentation process was used as a model to optimize the MSPD extraction method.

#### 3.3.1. Initial MSPD Assays

To determine the preliminary conditions that maximize the recovery of polyphenols, a series of initial experiments were performed, using the defatted cherry seed before fermentation as a model. In the initial trials, the effect of the dispersant nature, the sample–dispersant ratio (*w*/*w*), and the stirring mode were evaluated. For each of the phenolic extracts obtained, TFC and DPPH antioxidant capacity were determined.

To study the effect of the dispersant nature, silica, and diatomaceous earth were used as natural non-retentive abrasive material to ensure complete matrix disruption. In a similar study reported by Gómez-Mejía et al. [30], they showed that diatomaceous earth presents an excellent abrasive role with a high analyte-breaking capacity. Regarding the use of silica as a dispersant, previous studies described by Daneshfar et al. [34] and Minuti et al. [35] indicated that silica was characterized by an excellent matrix rupture capacity, presenting a high recovery of polyphenols. In addition, the natural origin of the dispersants promotes the application of an extraction procedure in the concept of green chemistry [36].

Another influential effect of weighing is the sample–dispersant mass relationship. According to Gómez-Mejía et al. [19], to evaluate the extraction efficiency, sample–dispersant ratios of 1/1 and 1/4 (*w*/*w*) were studied, setting the sample amount at 0.1000 g. Finally, in the initial MSPD trials, the effect of using vortex (402.48× *g*) or ultrasonic bath (720 W, 3.1% amplitude) as stirring modes on phenolic recovery was also evaluated.

**Figure 1:**
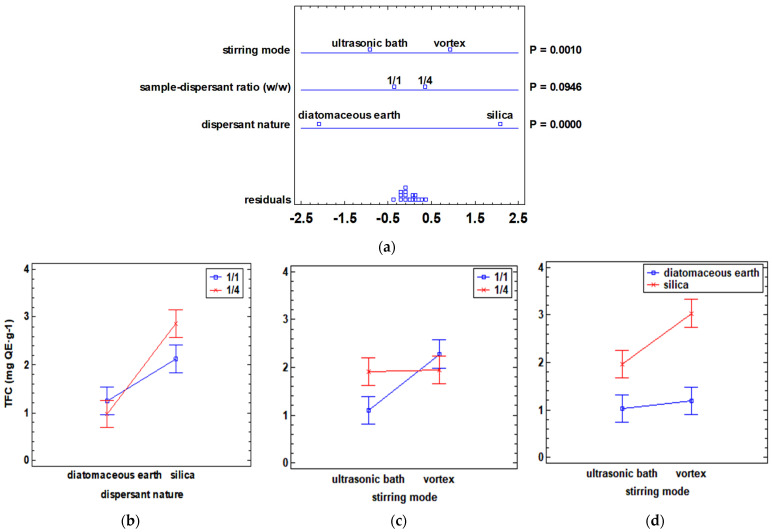
Multivariate ANOVA and interaction plots showing the influence of dispersant nature, sample–dispersant ratio (*w*/*w*), and stirring mode on TFC (mg QE·g^−1^) response: (**a**) multivariate ANOVA plot; (**b**) interaction graph between the nature of the dispersant and sample–dispersant ratio (*w*/*w*); (**c**) interaction graph between stirring mode and sample–dispersant ratio (*w*/*w*); (**d**) interaction graph between stirring mode and nature of dispersant. TFC is expressed as mg QE·g^−1^ of dry sample. QE: quercetin equivalent. *p*-values (P) lower than 0.05 indicate a statistically significant effect at a 95% confidence level.

On the one hand, the effect of each of the factors on the TFC response was studied, and a multifactorial ANOVA analysis was performed. The multivariate ANOVA plot and the interaction plots between each of the factors are shown in Figure 1.

The multivariate plot for the TFC response (Figure 1a) shows that stirring mode and the dispersant nature performed a significant impact (*p*-value < 0.05) on the TFC response. In terms of the nature of the dispersant, the highest TFC values were obtained when silica was used. Additionally, the interaction between the crossover effect of the sample–dispersant ratio and the nature of the dispersant was found to be significant (*p*-value < 0.05). Silica even presented a different behavior when it was in a 1/1 (*w*/*w*) or 1/4 (*w*/*w*) ratio with respect to the sample, which is not observed with diatomaceous earth (Figure 1b). Thus, the use of silica in a ratio of 1/4 (*w*/*w*) presented a predominantly higher flavonoid extraction. Furthermore, the interaction between the nature of the dispersant and the stirring mode was not significant (*p*-value > 0.05), although the highest TFC values were observed using vortex stirring combined with silica (Figure 1d). As for the interaction between the sample–dispersant ratio and stirring mode, it turned out to be non-significant in the TFC response (*p*-value > 0.05). The highest TFC values were achieved with the combination of vortex and 1/1 (*w*/*w*) sample–dispersant ratio (Figure 1c).

**Figure 2:**
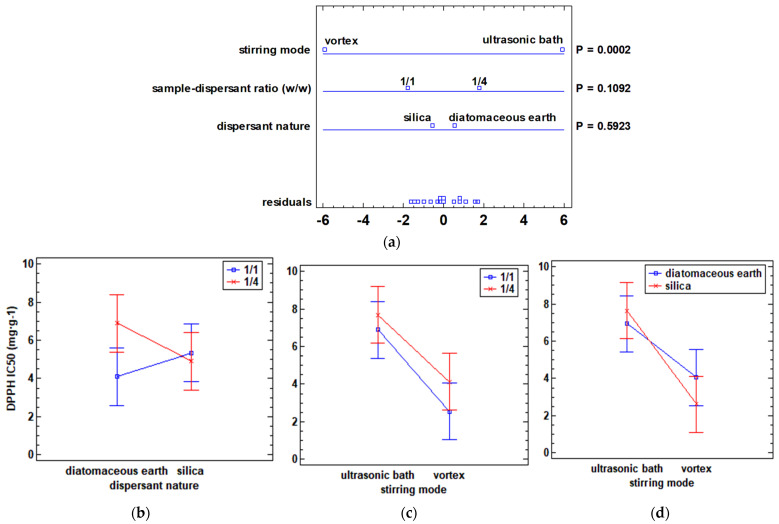
Multivariate ANOVA and interaction graphs showing the influence of dispersant nature, dispersant–sample ratio (*w*/*w*), and stirring mode on DPPH (IC_50_) response: (**a**) multifactorial ANOVA plot; (**b**) interaction graph between the nature of the dispersant and sample–dispersant ratio (*w*/*w*); (**c**) interaction graph between stirring mode and sample–dispersant ratio (*w*/*w*); (**d**) interaction graph between stirring mode and nature of dispersant. IC_50_: concentration needed to reduce 50% of DPPH. IC_50_ is expressed as mg·g^−1^ of dry sample. *p*-values (P) lower than 0.05 indicate a statistically significant effect at a 95% confidence level.

On the other hand, the effect of the studied experimental factors (nature of the dispersant, sample–dispersant ratio (*w*/*w*), and stirring mode) on the DPPH antioxidant capacity response was also evaluated. A multifactorial analysis of variance was performed, and the multivariate ANOVA plot and the interaction plots between each factor are shown in Figure 2.

As for the DPPH antioxidant capacity response, the only factor that was statistically significant (*p*-value < 0.05) was stirring mode. A significant impact on its response was observed when the ultrasonic bath or vortex was used as a stirring method. Thus, the highest values of antioxidant capacity (IC_50_ values lower) were obtained with the vortex stirring mode. To evaluate the possible interactions between the different variables, the interaction graphs shown in Figure 2b–d were represented. The only interaction statistically significant (*p*-value < 0.05) was the combination of vortex and sample–dispersant ratio (*w*/*w*). The interaction graph between the nature of the dispersant and the sample–dispersant ratio (*w*/*w*) (Figure 2b) revealed that there were no significant differences (*p*-value > 0.05) between silica when using one or another sample–dispersant ratio but that the lowest IC_50_ values (higher antioxidant capacity) were obtained with the ratio 1/4 (*w*/*w*). Furthermore, the interaction plots shown in Figure 2c,d showed that the lowest IC_50_ values were obtained with the combination of vortex and sample–dispersant ratio 1/1 (*w*/*w*) as well as with the combination of vortex and silica as a dispersant.

Therefore, according to the results obtained for each of the evaluated responses, the preliminary MSPD extraction conditions were established using silica as a matrix dispersant and vortex as a stirring method.

#### 3.3.2. Experimental Design and Optimization

Based on the aforementioned results and to obtain the optimal extraction conditions, a two-level factorial experimental design with three replicates at the central point was performed for the three selected experimental factors (sample–dispersant (*w*/*w*), ethanol–water ratio (*v*/*v*) in the extraction solution and vortex stirring time (min)), with a total of 11 experiments, as described in Section 2.3. The responses obtained for total polyphenol content (TPC), total flavonoid content (TFC), and antioxidant capacity DPPH (IC_50_) for each of the planned experiments are shown in Table 2.

Furthermore, Table 3 shows the values of the parameters that fit the experimental data to Equation (1) for each response evaluated. The mathematical models obtained for TPC, TFC, and DPPH responses presented satisfactory determination coefficients (R^2^) between 0.926 and 0.999. Accordingly, the standard errors of estimation (SSE) for the experimental responses TPC, TFC, and DPPH were acceptable (0.009–1.424). This indicated an adequate fit of the experimental data to the model fit.

Based on the values of the coefficients obtained for the mathematical models (Table 3), it could be deduced that the experimental factor ethanol–water ratio (*v*/*v*) ($\beta_{2}$) has the most relevant effect on the TPC, TFC, and DPPH responses, evidencing the strong impact of the solvent composition on the phenolic extraction. A positive correlation was observed between the ethanol–water ratio (*v*/*v*) ($\beta_{2}$) and the TPC, TFC, and DPPH experimental responses. This fact could indicate that the increase in the organic proportion in the ethanol–water solution increases the content of both total polyphenols and flavonoids. However, this increases the IC_50_ value, decreasing the antioxidant capacity of the extract. A similar fact was observed by Carciochi et al. [37]. They demonstrated that by increasing the proportion of ethanol in the ethanol–water mixture as a solvent for extracting polyphenols in quinoa seeds, the extract was enriched in TPC and TFC, and there was also an increase in the IC_50_ value, so the antioxidant capacity of the extract was lower.

In the case of the TPC response, the experimental factor of ethanol–water ratio (*v*/*v*) was the only significant factor (*p*-value < 0.05) at a confidence level of 95%. For TFC response and DPPH antioxidant activity, all the factors and their interactions with each other were not statistically significant (*p*-value > 0.05). Regarding sample–dispersant (*w*/*w*), increasing the proportion of dispersant ($\beta_{1}$) sharply decreased the IC_50_ (increasing the antioxidant capacity of the extract) and the content of total flavonoids while increasing the total polyphenol content. Considering the stirring time ($\beta_{3}$), higher values promoted an increase in the IC_50_ value, thus decreasing the antioxidant capacity of the extract, and the TFC significantly raised in turn (*p*-value < 0.05).

**Figure 3:**
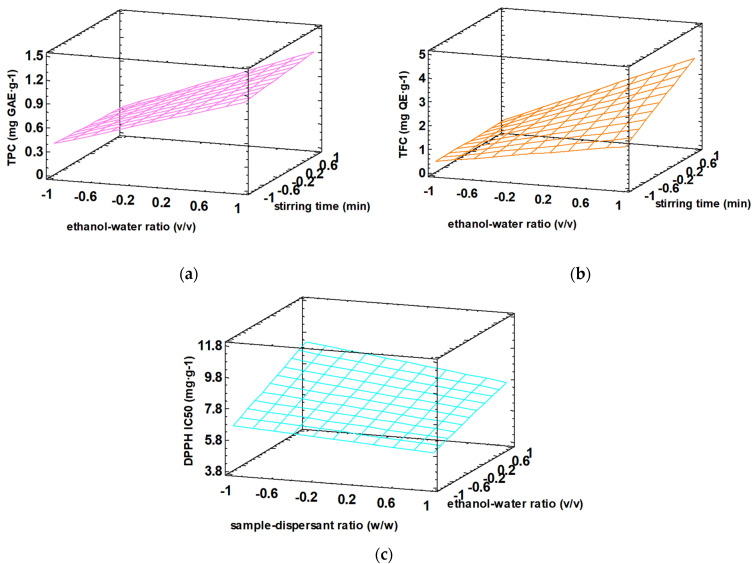
Normalized estimated responses surfaces of (**a**) TPC (mg GAE·g^−1^), (**b**) TFC (mg QE·g^−1^), and (**c**) antioxidant activity DPPH IC_50_ (mg·g^−1^). The experimental factor of sample–dispersant ratio was fixed to 1/1 (*w*/*w*) for TPC and TFC responses, while the experimental factor of stirring time was established to 5 min for the DPPH response. TPC, TFC, and IC_50_ values are expressed on a dry basis. GAE: gallic acid equivalent, IC_50_: concentration needed to reduce 50% of DPPH, and QE: quercetin equivalent.

To explain the effect of each experimental factor on the total polyphenol content, total flavonoid content, and DPPH scavenging activity responses, estimated response surfaces were obtained, resulting in three-dimensional graphs, as shown in Figure 3.

The normalized response surfaces obtained for total polyphenol content (Figure 3a) and total flavonoid content (Figure 3b) were quite similar. A small distortion of the plane was observed in the case of TFC response, suggesting a possible interaction between the factors. Nevertheless, the normalized response surface for DPPH (Figure 3c) was completely different. The highest TPC and TFC values were observed for 80:20 (*v*/*v*) ethanol–water ratio and 10 min of vortex stirring (Figure 3a,b), while the lowest IC_50_ value (i.e., the highest antioxidant capacity of the phenolic extract) (Figure 3c) was obtained for 1/5 (*w*/*w*) sample–dispersant ratio and 20:80 (*v*/*v*) ethanol–water proportion.

The measurements of TPC and TFC of the phenolic extracts showed the same optimal MSPD extraction conditions, while they were different for the antiradical activity (DPPH) of the phenolic extracts. Therefore, a response surface analysis (RSA) was carried out with the aim of setting the MSPD optimal extraction conditions for each individual response (TPC, TFC, and IC_50_ value). Hence, the optimization criteria were to maximize both TPC and TFC and to minimize the IC_50_ value. The optimal extraction conditions for each of the evaluated responses are shown in Table 4. Accordingly, the optimum extraction conditions were the same for total polyphenol content and total flavonoid content, implying a sample–dispersant ratio of 1/1 (*w*/*w*), an ethanol–water ratio of 80/20 (*v*/*v*), and a stirring time in a vortex for 10 min. Under these conditions, desirability function values of 0.9964 and 0.9327 were achieved for TPC and TFC, respectively. Meanwhile, for the DPPH antioxidant capacity, they account for the use of 1/5 (*w*/*w*) sample–dispersant ratio, 20:80 (*v*/*v*) ethanol–water ratio, and 5 min of stirring time. Under these extraction conditions, the individual desirability function value of the DPPH response was 0.8200. Given the heterogeneity of the optimal extraction conditions, it was necessary to resort to a multiple response analysis (MRA), which made it possible to find a unique combination of experimental factors and simultaneously optimize the three responses studied (Table 4). The optimization criterion selected was to maximize the total desirability function. As optimal compromise conditions for the simultaneous extraction of phenolic MSPD, a sample–dispersant ratio of 1/1 (*w*/*w*), ethanol–water ratio of 80:20 (*v*/*v*), and a vortex stirring time of 10 min were selected. Under these conditions, a high value of the total desirability function (0.9134) was reached.

The experimental responses obtained under optimal MSPD extraction conditions established by the multiple response analysis (MRA) were compared with those predicted by the model, using the two-tailed Student’s *t* at a confidence level of 95% (Table 5).

The data collected in Table 5 show that the predicted values and the experimental values are not significantly different (***t*_calc_** < ***t*_exp_**), at a 95% confidence level, for the TPC and TFC responses. In the case of DPPH antioxidant capacity, there is a significant difference (***t*_calc_** > ***t*_exp_**). However, the lower the IC_50_ value, the greater the antioxidant capacity of the extract, so it is of interest that the experimental value found for the IC_50_ was significantly lower than that predicted by the model. Accordingly, the experimental results obtained under the optimal conditions established for the extraction of bioactive polyphenols MSPD present an adequate correlation with the values predicted by the model through the design of experiments, being within the confidence interval of the experimental determination.

Ultimately, the optimal MSPD extraction conditions for the recovery of polyphenols from sweet cherry seeds were a sample–dispersant ratio of 1/1 (*w*/*w*), an ethanol–water ratio of 80:20 (*v*/*v*), and a vortex stirring time of 10 min.

### 3.4. Total Phenolic Content and Antioxidant Activities of Cherry Seed Extracts

**Figure 4:**
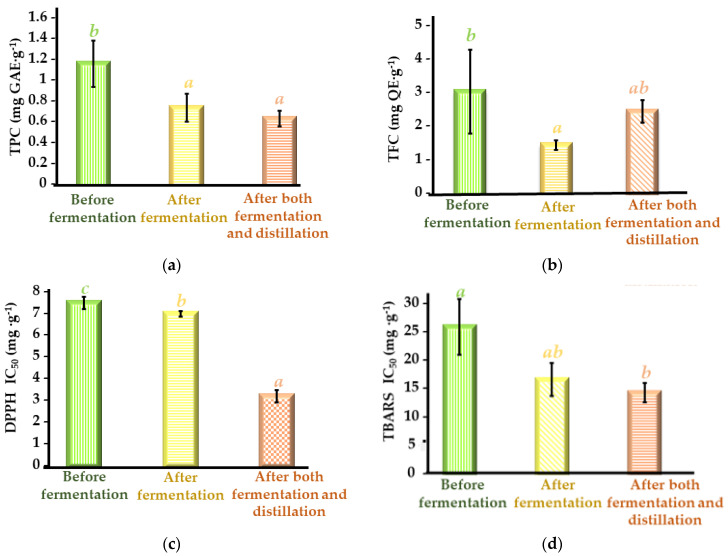
TPC, TFC, and antioxidant activity of defatted sweet cherry seed extracts before fermentation, after fermentation, and after both fermentation and distillation obtained under optimal MSPD extraction conditions: (**a**) TPC (mg GAE·g^−1^); (**b**) TFC (mg QE·g^−1^); (**c**) antioxidant capacity DPPH radical scavenging IC_50_ (mg·g^−1^); (**d**) antioxidant capacity TBARS assay IC_50_ (mg·g^−1^). IC_50_: concentration required to reduce 50% of DPPH. All data are expressed on a dry basis and as the mean ± standard deviation (*n* = 3). Mean values with different letters indicate significant differences with *p*-value < 0.05, according to one-way ANOVA and Fisher’s LSD test. DPPH IC_50_ trolox = 2.5 ± 0.1 mg·mL^−1^. TBARS IC_50_ = 11.6 ± 0.2 mg·mL^−1^.

Once the optimal MSPD extraction conditions were determined, the total polyphenol content and total flavonoid content, as well as the antioxidant capacity (expressed as IC_50_), were determined for all the sweet cherry defatted seed residues generated during the cherry liquor processing in establishing the bioactive potential of each of the extracts. For this purpose, the procedures explained in Section 2.5, Section 2.6 and Section 2.7 were followed, and the results obtained are shown in Figure 4.

The defatted seeds (before fermentation) were characterized by a TPC of 1.37 ± 0.08 mg GAE·g^−1^ (Figure 4a) and a TFC of 3 ± 1 mg QE·g^−1^ (Figure 4b). These results were similar to those reported by Yüksekkaya et al. [38], who reported that sweet cherry seeds had a TPC of 2 mg GAE·g^−1^ and a TFC of 1.8 mg QE·g^−1^. Furthermore, Hu et al. [39] also determined that sweet cherry seeds from four different crops were characterized by a TPC of 0.87–1.73 mg GAE·g^−1^ and a TFC of 0.31–0.51 mg QE·g^−1^. Another important characteristic of the seeds is their antioxidant activity, given that this property is largely related to their phenolic content and estimates the ability by which the active compounds in cherry seeds can protect cells from oxidative stress [40,41]. Considering that antioxidant activity can be exerted by several mechanisms, two proton atom transfer methods were used, i.e., DPPH assay and TBARS assay. Furthermore, it is worth mentioning that the TBARS test is an in vitro methodology whose results are of relevance in the agri-food approach as a lipid peroxidation model [42]. All the phenolic extracts studied (Figure 4c,d) presented an appreciable antioxidant activity (DPPH IC_50_ of 3.2–7.4 mg·g^−1^ and TBARS IC_50_ of 14–26 mg·g^−1^). The positive control, trolox, presented a value of IC_50_ = 2.5 ± 0.1 mg·mL^−1^ for the DPPH antioxidant activity assay and a value of IC_50_ = 11.6 ± 0.2 mg·mL^−1^ for the TBARS lipid peroxidation assay. In addition, the trolox positive control showed that the coefficient of variation was) = 4% for DPPH and 2% for TBARS assay, showing a high reproducibility. Furthermore, the results obtained for the antioxidant capacity were compared with those obtained by Hu et al. [39] for cherry seed extracts, reporting DPPH free radical scavenging capacity IC_50_ values slightly higher than those reported herein (1.01 ± 0.80 mg·g^−1^).

In addition, TPC and TFC values of the seeds before fermentation, after fermentation, and after both fermentation and distillation (Figure 4a,b) were compared to study the variations in phenolic content during the processing of defatted seed residues to produce liquor. It was observed that after the fermentation process, the content of total polyphenols and the total flavonoid content were significantly (*p*-value < 0.05) reduced in comparison to the seed before fermentation. The seed before fermentation presented a TPC value of 1.37 ± 0.08 mg GAE·g^−1^ and a TFC value of 3 ± 1 mg QE·g^−1^, while the seed after the fermentation process exhibited a TPC value of 0.98 ± 0.07 mg GAE·g^−1^ and a TFC value of 1.4 ± 0.1 mg QE·g^−1^. Vasile duff et al. [43] also observed that the phenolic content of plum by-products decreased drastically after the alcoholic fermentation process, which could be attributed to the polymerization of phenolics released by oxidation enzymes, activated in response to stress induced in the microorganism due to nutrient depletion. Furthermore, Jericó-Santos et al. [44] demonstrated that during the alcoholic fermentation process, a membrane diffusion mechanism of phenolic compounds from one matrix to another occurs. A concentration gradient is generated by observing the loss of phenolic compounds in one matrix and, at the same time, an increase in the content of phenolic compounds in another matrix. Therefore, the decrease in the phenolic content (TPC and TFC) of the defatted cherry seed (Figure 4a,b) during the processing to obtain liquor could be due to the concentration of phenolic compounds in the seed migrating to the pulp or other by-products used to obtain the cherry liquor, enriching the obtained liquor in phenolic compounds.

Furthermore, another interesting fact was shown by Dey et al. [45], revealing that during the fermentation process, extracellular enzymes are produced and used simultaneously for the release of phenolic compounds from the solid matrix, with the production of new bioactive compounds through the secondary metabolism of microorganisms. Hence, the appearance of new compounds that can positively affect antioxidant capacity is feasible. This is correlated with that shown in Figure 4c,d, where an increase in the antioxidant capacity of the cherry seed (lower IC_50_ value) is observed after fermentation. Before fermentation, the seed showed an antioxidant capacity to eliminate free DPPH radicals (IC_50_ = 7.4 ± 0.3 mg·g^−1^), compared to the seed after fermentation, which displayed an IC_50_ value of 6.9 ± 0.1 mg·g^−1^, which differed significantly (*p*-value < 0.05) from the previous one. A similar behavior was observed regarding TBARS antioxidant capacity. An IC_50_ value of 26 ± 5 mg·g^−1^ was observed in defatted cherry seeds before the fermentation process compared to the seed after fermentation, for which the value of IC_50_ decreased to 17 ± 3 mg·g^−1^, meaning an increase in the anti-lipid peroxidation capacity.

With respect to the distillation process, it was observed that the phenolic content (Figure 4a) of the seeds after both fermentation and distillation (0.7 ± 0.1 mg GAE·g^−1^) decreased with respect to the cherry seeds after fermentation (0.98 ± 0.07 mg GAE·g^−1^), although not in a statistically significant way (*p*-value > 0.05). This may be due to the fact that in the distillation process, the seed is subjected to high temperatures that can degrade labile components, such as phenolic compounds [24].

Regarding the total flavonoid content (Figure 4b), an increase in the TFC value was observed in the cherry seed after both fermentation and distillation (2.3 ± 0.3 mg QE·g^−1^) with respect to the seed after fermentation (1.4 ± 0.1 mg QE·g^−1^), although this difference was not statistically significant (*p*-value > 0.05). It is well known that flavonoids are sensitive to temperature effects; for example, glycosylated flavonoids are more resistant to temperature than aglycon flavonoids. In addition, the degradation process that flavonoids undergo at high temperatures makes the formation of new flavonoids possible; for example, quercetin in the presence of oxygen and high temperatures favors the formation of four new flavonoids from it. Therefore, it is possible that during the distillation process, new flavonoids are formed in the seeds, and therefore, their content increases [46].

Regarding the DPPH and TBARS antioxidant capacity (Figure 4c,d) of the seed extracts after both fermentation and distillation (DPPH IC_50_ = 3.2 ± 0.3 mg·g^−1^; TBARS IC_50_ = 14 ± 2 mg·g^−1^), a significant decrease in the IC_50_ value was observed with a consequent increase in the antioxidant activity with respect to the seed after fermentation (DPPH IC_50_ = 6.9 ± 0.1 mg·g^−1^; TBARS IC_50_ = 17 ± 3 mg·g^−1^). During the distillation process, the degradation of phenolic compounds, such as flavonoids, occurs, favoring the formation of new degradation products that could have a higher antioxidant activity.

In conclusion, sweet cherry defatted seed phenolic extracts before fermentation exhibited the highest TPC and TFC values, but those with the best antioxidant activities were the defatted seed extracts obtained after both fermentation and distillation processes. The increase in antioxidant activity after both fermentation and distillation may be due to the presence of other bioactive compounds, such as carbohydrates and proteins [8]. In addition, as previously reported, during the fermentation process, extracellular enzymes are produced that can lead to the production of new bioactive compounds through the secondary metabolism of the microorganisms, which could positively affect their antioxidant capacity [45]. Hence, the employment of chromatographic methods is required to elucidate the phenolic profile of the seed extracts and to correlate their phenolic composition with their antioxidant activity.

### 3.5. Phenolic Profile of Cherry Seed Extracts

Spectrophotometric methods are suitable for determining the content of total polyphenols and total flavonoids, as well as the antioxidant capacity of phenolic extracts; however, for an individual phenolic characterization with adequate sensitivity and selectivity in the analysis, it is necessary to use chromatographic techniques [30]. In this study, phenolic extracts obtained under optimal MSPD extraction conditions were compared from three different defatted cherry (*Prunus avium* L.) seed residues generated at different stages of cherry liquor production (before fermentation, after fermentation, and following fermentation and distillation). The phenolic profile of all cherry seed residues was investigated using a non-targeted approach by HPLC-ESI-MS/MS, leading to the identification of 13 phenolic compounds (Table 6). Accordingly, nine phenolic acids, including four hydroxybenzoic acids, as follows: gallic acid (peak 1), 2,3-dihydroxybenzoic acid (peak 2), vanillic acid (peak 5), and syringic acid (peak 7), and five hydroxycinnamic acids, namely neochlorogenic acid (peak 3), chlorogenic acid (peak 4), caffeic acid (peak 6) *p*-coumaric acid (peak 8), and *trans*-ferulic acid (peak 9) acid. Flavonoids were found to be minor, identifying low-molecular-weight flavonols such as quercetin (peak 12) and kaempferol (peak 13), as well as two rutinosides (peaks 10–11). Previous work has reported the presence of the above flavonols, hydroxycinnamic, and hydroxybenzoic acids in cherry seeds, both as aglycones and glycoside-derived forms [9,47]. However, the low proportion of glycosidized polyphenols reported in this study could be due to the previous recovery of the fatty acid-rich oil from the seed, in which temperatures around 69 °C are employed, favoring the cleavage of the *O*-glycoside bond [31].

In addition, a non-phenolic compound was detected in extracts obtained from residual sweet cherry seeds after fermentation and distillation. This peak appeared at 6.8 min, with a precursor [M-H]^−^ ion at *m*/*z* 456.151211 and MS/MS fragments at *m*/*z* 391.2956, 323.0798, 263.0537, 221.0551, 161.0341, and 119.0294, leading to a molecular formula of C_20_H_27_NO_11_. According to the literature, the fragment at *m*/*z* 323 resulted from the neutral loss of a disaccharide [M-H-133]^−^, tentatively identifying this compound as amygdalin [47,48]. This is a cyanogenic diglycoside (D-mandelonitrile-*β*-D-gentiobioside; syn: D-mandelonitrile-*β*-D-glucosido-6-*β*-glucoside) found mainly in the seeds of the *Rosaceae* species, which includes *Prunus avium* L., among others [49]. Amygdalin and other cyanogenic glycosides are mainly released when the seeds are processed [47]. Thus, this compound has only been detected in the seed extracts obtained after both fermentation and distillation and not in those prior to the fermentation process. Amygdalin can be hydrolyzed, generating prunasin, glucose, and mandelonitrile under the action of *β*-glucosidase, which is stored in the plant cells compartments and presents in the human small intestine and finally decomposes into benzaldehyde and hydrocyanic acid (HCN) [49,50]. Even though amygdalin itself is not considered a toxic compound, HCN is a poisonous substance [51]. Therefore, its use was restricted by the Food and Drug Administration (FDA). Nowadays, it is well known to be harmful to the human body when levels are exceeded in oral, intramuscular, or intravenous administration [52]. Particularly, the consumption of 50 bitter almonds in a short period of time can be a lethal dose for an adult, and a dose of 5–10 bitter almonds can be poisonous to a child [51]. It is possible to reduce their toxicity levels to minimum levels under the application of detoxification methods such as drying, roasting, thermal, immersion, or ultrasound treatments [53,54].

Despite the toxicity of amygdalin, there is currently some controversy about the potential human health benefits of amygdalin [51,55]. Several researchers have indicated in their in vitro cell culture studies the possible anti-cancer capacity of amygdalin [56,57,58]. Amygdalin induces apoptosis, which could inhibit cancer cell proliferation and survival [56]. Sushma et al. [59] have demonstrated that the cytotoxic activity of amygdalin against HeLa cancer cells was relatively high. Kitic et al. [60] reported that amygdalin has an antiproliferative effect on CAF-7 cells, increasing early and late cell apoptosis and arresting the cell cycle to control breast cancer. Kwon et al. [61] demonstrated that amygdalin is able to induce apoptosis in human promyelocytic leukemia cells (HL-60). However, in vivo studies have shown that the effects of amygdalin are not homogeneous, and the results are ambiguous, which has contributed to deepening the gap between proponents and detractors of amygdalin as a useful cancer treatment [52]. Therefore, it is necessary to broaden the knowledge about this cyanogenic glucoside and its possible effects on the human body to clarify its health behavior.

Other preclinical studies indicated the pharmacological potential of amygdalin, including anti-inflammatory, antinociceptive, and neurotrophic effects [52]. In this line, Gago-Lopez et al. [62] demonstrated that an amygdalin analog reduces the proliferative capacity of stimulated psoriasis-like keratinocytes and their inflammatory response in vivo and in vitro. Notwithstanding, these possible benefits of amygdalin are not currently known with adequate certainty, so it is necessary to scientifically explore this cyanogenic glucoside and perform in vivo and clinical studies as well as safety controls to evaluate its effects on human health [51]. In addition, another important factor to consider is the toxicity levels that amygdalin could present in its topical administration [63], since although no toxicity data have been reported on its dermal administration, this topical would be reabsorbed into the skin and cross the epidermal barrier, reaching the bloodstream, and could present appreciable levels of toxicity.

For all this previously mentioned, and considering the toxicity that amygdalin presents, in order to exploit the seed residue generated after fermentation and distillation in the food sector, it is first necessary to quantify its content in order to know the levels of toxicity that could be present in the human body. On the one hand, the development of methodologies that reduce their levels to minimum values, such as detoxification processes, could be of interest. On the other hand, it is necessary to carry out in vivo and in vitro studies, as well as safety methods, to know its behavior in the human body and if it presents any type of benefit.

The results of the quantification of the individual phenolic compounds of the cherry seed extracts are indicated in Table 7.

The main polyphenols present in the three defatted cherry seed extracts were 2,3-dihydroxybenzoic acid (38–84 μg·g^−1^), neochlorogenic acid (36–3.9 μg·g^−1^), caffeic acid (25.4–3 μg·g^−1^) and quercetin (16–5.8 μg·g^−1^). Furthermore, this corresponds with the data reported in the bibliography [9,16,39,64], where the main polyphenols that have been found in sweet cherry seeds were caffeic acid (10.4–11.53 μg·g^−1^), quercetin (3.9–13.9 μg·g^−1^) and neochlorogenic acid (23–39 μg·g^−1^). On the one hand, the content of phenolic acids in sweet cherry seed increased during processing to obtain liquor, although the increase was not significant (*p*-value > 0.05). The highest phenolic contents were observed in the cherry seed extracts after both fermentation and distillation (96 ± 9). These results were contrary to those observed in the *Folin–Ciocalteu* spectrophotometric assay for the determination of the total content of polyphenols (TPC). This fact could be attributed to the presence of monomeric, oligomeric, and polymeric polyphenols that are determined by the *Folin–Ciocalteu assay* (TPC), whereas only the low-molecular-weight compounds listed in Table 7 are covered by HPLC-ESI-QTOF-MS analysis. Hence, the cherry seed extracts before fermentation may have a high content of polymeric polyphenols that are not observed in the HPLC analysis [65]. Furthermore, it should be mentioned that the interference is likely from the sugars present in the seed, considering that these compounds are also capable of reacting with the *Folin–Ciocalteu* reagent. Since a high sugar content is expected in the extracts from cherry seeds before fermentation in comparison to that obtained after fermentation and after both fermentation and distillation, the total polyphenol content (TPC) is higher in the residue extracts before fermentation than in the others. Both facts could justify the discrepancy between chromatographic and spectrophotometric results. This difference between chromatographic and spectrophotometric methods for determining polyphenols was also observed by Maier et al. [65] in grape seed residues. According to the results shown in Figure 4a, the content of total polyphenols, including polymeric polyphenols, decreases progressively after the fermentation process due to the migration of these compounds from the seed to the pulp or other by-products of the fruit. Also, the high temperatures to which the defatted seeds after both fermentation and distillation are subjected during the distillation process favor the degradation of polymeric polyphenols [24,45]. The determination of polyphenols using the *Folin–Ciocalteu* assay shows the most information on the content of total polyphenols since oligomeric and/or polymeric polyphenols are not detected in HPLC analyses. On the other hand, the content of total flavonoids in the defatted seed waste progressively decreased during the processing to obtain cherry liquor. A significant decrease (*p*-value < 0.05) was observed from the initial cherry seed extracts (16 ± 2) to the extracts after fermentation (8 ± 2) and after both fermentation and distillation (5.8 ± 0.1). These results were similar to those observed in the spectrophotometric assays to determine the total flavonoid content (TFC). As mentioned previously, during the seed fermentation process, flavonoids migrate to other parts of the fruit, such as the pulp or other residues that are used during the processing to obtain liquor [23,45]. According to the chromatographic analysis, the flavonoid content is reduced, although not significantly, from the cherry seed extracts after fermentation (8 ± 2) to the extracts after fermentation and distillation (5.8 ± 0.1). However, the opposite is observed in the results from the spectrophotometric tests for determining the total flavonoid content, which may be because during the distillation process, the formation of new flavonoids with a more convoluted structure, such as tannins, is favored. Tannins are not determined by HPLC analysis, but they present a positive response in the TFC assays [46].

**Figure 5:**
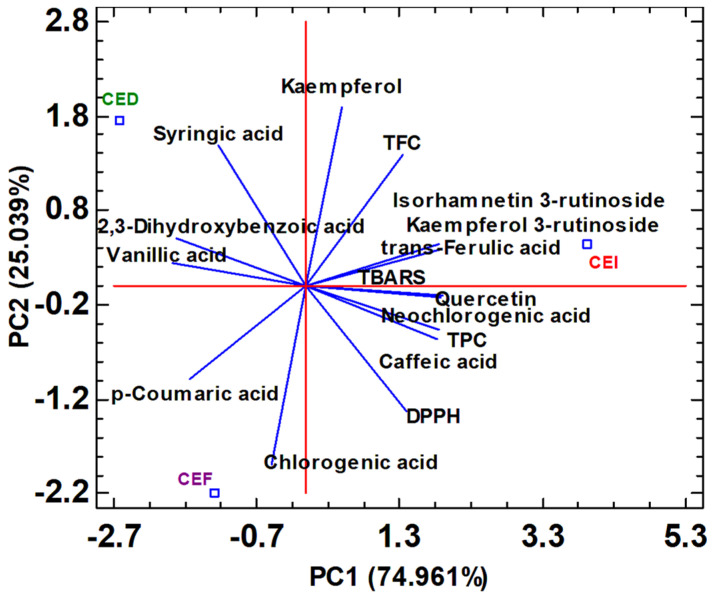
Principal component analysis biplot of the simultaneous evaluation of scores (CEI: defatted cherry seed before fermentation, CEF: defatted cherry seed after fermentation, and CED: defatted cherry seed after both fermentation and distillation) and loadings (individual polyphenols extracted and total parameters estimated under optimum extraction conditions). TPC: total polyphenol content, TFC: total flavonoid content, DPPH: scavenging activity of extracts against 1,1-diphenyl-2 picrylhydrazyl free radical, expressed as IC_50_ mg extract·g^−1^ of dry sample and TBARS: thiobarbituric acid reactive substances, expressed as IC_50_ mg extract·g^−1^ of dry sample.

With the purpose of easily studying and correlating the antioxidant capacity (DPPH and TBARS) and the phenolic indexes (total polyphenol content and total flavonoid content) with the phenolic profile of the samples (chromatographic data), a multivariate analysis by principal component analysis (PCA) was carried out. This chemometric tool made it possible to simultaneously study the variation in the phenolic content of extracts of defatted sweet cherry seeds before fermentation, after fermentation, and after both fermentation and distillation. The results of PCA analysis are shown in Figure 5.

Two principal components with eigenvalues greater than or equal to 1, accounting for 100% of the total data variability, were extracted. The first principal component (PC1) accounted for 74.961% of the total variability, and the second principal component (PC2) explained 25.039% of the total variability. For this purpose, the data matrix was decomposed into matrices of scores (sample coordinates) and loadings (polyphenols, TPC, TFC, DPPH, and TBARS antioxidant capacity), which provide information on the samples and factors, respectively.

A difference in phenolic content was observed in the defatted seed extracts during the processing stages. It was detected that defatted seed cherry before fermentation was characterized by a high content of kaempferol 3-rutinoside, isorhamnetin 3-rutinoside, quercetin, and neochlorogenic acid. Kim et al. [66] demonstrated that these phenolic compounds were the major ones found in sweet cherries. They present antineurodegenerative activity, also acting as a suitable source of biofunctional phytochemicals in the diet. In addition, numerous studies have pointed out that kaempferol-3-rutinoside has multiple benefits, such as antioxidant, anti-inflammatory, antimicrobial, anti-cancer, prevention of heart disease, neurological diseases, antidiabetic, anti-osteoporosis, anti-estrogenic, analgesic, and hypoallergenic. Moreover, this compound is also a potent inhibitor of hyaluronidase enzymes and, therefore, also shows anti-aging properties [67]. Neochlorogenic acid exhibits anti-inflammatory, antibacterial, antioxidant, and anticarcinogenic properties [68]. In addition, the presence of high quercetin content in the seed residue extracts could make them excellent as antidiabetic, anti-cancer, and antioxidant, among others [69]. Also, the lowest levels of antioxidant capacity (high IC_50_ values) were shown in this residue. Defatted cherry seed after fermentation presented a high positive correlation with chlorogenic acid, and the seed obtained after both fermentation and distillation process had a positive correlation with dihydroxybenzoic acid and vanillic acid. The multiple benefits of chlorogenic acid on human health are also well known. Including its antidiabetic, anti-cancer, anti-inflammatory, and anti-obesity effects, it may provide a non-pharmacological and non-invasive approach to the treatment or prevention of some chronic diseases [70].

In addition, the best DPPH and TBARS antioxidant activity was observed in this type of cherry seed. Therefore, the high antioxidant capacity could be related to the content of 2,3-dihydroxybenzoic acid and vanillic acid. It is currently well known that these polyphenols have outstanding antioxidant, anti-inflammatory, and neuroprotective properties [71,72]. In the biplot represented in Figure 5, it was observed that the defatted cherry seed before fermentation presented a high content of neochlorogenic acid, while the seed after fermentation process exhibited a high concentration of its respective isomer, chlorogenic acid. Data in the literature indicated that neochlorogenic acid is more stable than chlorogenic acid, so it is more commonly observed in the seeds after fermentation. However, during the subsequent process of removal of fatty acids, due to the high temperatures used in this process, it is possible that the bonds are weakened, and the formation of chlorogenic acid is easily favored [73]. It was also noticed that the defatted cherry seeds before fermentation showed a high content of caffeic acid, which was negatively correlated with vanillic acid, the main polyphenol presented in cherry seed after both fermentation and distillation. This could be due to the fact that the high temperatures during the distillation process favor the oxidative degradation process of caffeic acid into vanillic acid [74]. In addition, in the PCA graph, it was also observed that the individual polyphenols presented in the defatted cherry seed before fermentation disappeared during the fermentation and subsequent distillation process, giving rise to the appearance of new polyphenols, such as chlorogenic acid, vanillic acid, *p*-coumaric acid or the increase in 2,3-dihydroxybenzoic acid content. This could be attributed to the enzymatic degradation process during fermentation and the high temperatures involved in the distillation process [74].

Therefore, the presence of high contents of kaempferol 3-rutinoside, isorhamnetin 3-rutinoside, quercetin, and neochlorogenic acid in the defatted sweet cherry seed extracts before fermentation could make possible their application as active ingredients in the cosmetic, food, pharmaceutical, and/or nutraceutical industries due to their antioxidant, anti-inflammatory, anticarcinogenic, and neuroprotective activities [8,66,67,68,69]. Furthermore, these phenolic compounds could be used in the preservation of perishable products of microbial attack due to their exclusive antioxidant and free radical scavenger properties [75]. The application of these antioxidant compounds in cosmetics reduces oxidative damage, constituting a suitable alternative in therapy and prevention of premature aging. It also provides photoprotective action and helps in the treatment of sun-stressed skin by sensitive anti-inflammatory activity [76]. Regarding the defatted seed extracts obtained after fermentation, due to the high content of chlorogenic acid, its application in the food and nutraceutical industries may be interesting as a dietary supplement, as well as in the pharmaceutical and cosmetic industry as an active ingredient in drugs and cosmetics [70]. Finally, the sweet cherry defatted seed extracts obtained after both fermentation and distillation may have potential applications in the cosmetic industry as a potential active ingredient due to the high antioxidant properties attributed to 2,3-dihydroxybenzoic and vanillic acids. Notwithstanding, the presence of the cyanogenic glycoside amygdalin restricts their potential use. As previously reported, toxicity is the main limitation that amygdalin presents when its levels are exceeded in the blood during intramuscular, intravenous, or oral administration. Therefore, it is necessary to determine the amygdalin content of the cherry seed extract after both fermentation and distillation and subsequently apply in vitro and in vivo safety studies to evaluate whether the extract presents any type of health risk. The application of amygdalin detoxification methods could also be developed and evaluated to reduce its levels to minimum values and, therefore, reduce its toxicity so that its possible industrial application can be studied. Furthermore, the toxicity of amygdalin has been demonstrated in oral or intravenous administration, but its level of toxicity in dermal administration is not known [53,58]. Several studies [58,77] indicated that amygdalin is capable of treating skin diseases such as psoriasis. Therefore, a possible industrial output of the sweet cherry seed extracts after both fermentation and distillation as an ingredient in the production of cosmetic topicals for the skin. In this line, safety and quality tests are necessary, as a priority, to ensure its non-toxicity to the human body. As well as in vitro and in vivo studies that support its possible benefits on the skin without being harmful to human health.

## 4. Conclusions

In the present work, a poorly characterized and explored bioresidue, namely defatted sweet cherry seeds generated during the production of cherry liquor (defatted sweet cherry seed before fermentation, after fermentation, and after both fermentation and distillation) and subsequent removal of fatty acids, is examined. Therefore, not only are pressed cherry seeds being looked at as a source of polyphenols but also a new approach to continuous waste valorization processes within the agricultural industry, which is presented here. For that aim, a non-conventional and environmentally friendly MSPD method has been developed and optimized using ethanol–water 80:20 (*v*/*v*), silica in a 1/1 ratio to the sample ratio, and 10 min of vortex stirring. The initial defatted cherry seed extracts (before fermentation) showed the highest TPC and TFC values. However, the maximum DPPH and TBARS antioxidant capacity was observed in the cherry defatted seed extracts obtained after both fermentation and distillation. The phenolic characterization by HPLC-ESI-MS/MS has made it possible to correlate the phenolic chemical composition of each of the obtained seed residue extracts with the effect of processing. The extract of cherry seed after fermentation with high chlorogenic and *p*-coumaric acids content could be used in industrial applications that require a great anti-inflammatory and antimicrobial potential, while the cherry seed extracts obtained after both fermentation and distillation, with a high content of 2,3-dihydroxybenzoic and vanillic acids and the presence of amygdalin, requires greater scientific knowledge and development of methods that take into consideration its toxicity levels for possible future exploitation of this type of by-product.

## Figures and Tables

**Table 1:** Plan of experiments for factorial design to extract polyphenols by MSPD extraction.

Experiment	Factors		
	Sample–Dispersant Ratio (*w*/*w*) $\beta_{1}$	Ethanol–Water Ratio (*v*/*v*) $\beta_{2}$	Vortex Stirring Time (min) $\beta_{3}$
1	1/1 (−1)	20:80 (−1)	5 (−1)
2	1/5 (1)	20:80 (−1)	5 (−1)
3	1/1 (−1)	80:20 (1)	5 (−1)
4	1/1 (−1)	20:80 (−1)	10 (1)
5	1/3 (0)	50:50 (0)	7.5 (0)
6	1/5 (1)	20:80 (−1)	10 (1)
7	1/3 (0)	50:50 (0)	7.5 (0)
8	1/5 (1)	80:20 (1)	10 (1)
9	1/1 (−1)	80:20 (1)	10 (1)
10	1/5 (1)	80:20 (1)	10 (1)
11	1/3 (0)	50:50 (0)	7.5 (0)

$\beta_{1}$, $\beta_{2}$, and $\beta_{3}$ are the linear coefficients of sample–dispersant ratio (*w*/*w*), ethanol–water ratio (*v*/*v*), and stirring time in the vortex. Normalized values of each factor level are shown in parentheses.

**Table 2:** Experimental responses obtained from the modeling set to extract polyphenols by MSPD extraction.

Experiment	Factors			Responses		
	Sample–Dispersant Ratio (*w*/*w*) $\beta_{1}$	Ethanol–Water Ratio (*v*/*v*) $\beta_{2}$	Vortex Stirring Time (min) $\beta_{3}$	TPC (mg GAE·g^−1^)	TFC (mg QE·g^−1^)	DPPHIC_50_ (mg·g^−1^)
1	1/1 (−1)	20:80 (−1)	5 (−1)	0.344	0.741	5.122
2	1/5 (1)	20:80 (−1)	5 (−1)	0.441	0.157	4.369
3	1/1 (−1)	80:20 (1)	5 (−1)	1.078	1.588	10.816
4	1/1 (−1)	20:80 (−1)	10 (1)	0.358	0.480	8.191
5	1/3 (0)	50:50 (0)	7.5 (0)	0.494	1.854	4.389
6	1/5 (1)	20:80 (−1)	10 (1)	0.341	0.545	7.485
7	1/3 (0)	50:50 (0)	7.5 (0)	0.450	1.497	4.372
8	1/5 (1)	80:20 (1)	10 (1)	1.118	1.812	7.190
9	1/1 (−1)	80:20 (1)	10 (1)	1.272	4.451	7.734
10	1/5 (1)	80:20 (1)	10 (1)	1.171	3.013	8.184
11	1/3 (0)	50:50 (0)	7.5 (0)	0.445	1.357	4.331

$\beta_{1}$, $\beta_{2},$ and $\beta_{3}$ are the linear coefficients of dispersant–sample ratio (*w*/*w*)), ethanol–water ratio (*v*/*v*)), and vortex stirring time. Normalized values of each factor level are shown in parentheses. TPC, TFC, and IC_50_ values are expressed on a dry basis. IC_50_: concentration needed to reduce 50% of DPPH, GAE: gallic acid equivalent, and QE: quercetin equivalent.

**Table 3:** Values of the coefficients, correlation coefficient (R^2^), and standard error of estimation (SEE) obtained from the adjustment of the polynomial model described in Equation (1) for each of the responses evaluated.

Responses	Coefficients								
	$\beta_{0}$	$\beta_{1}$	$\beta_{2}$	$\beta_{3}$	$\beta_{12}$	$\beta_{13}$	$\beta_{23}$	R^2^	SEE
TPC (mg GAE·g^−1^)	0.7654	0.0024	**0.3944**	0.0201	−0.0176	−0.0319	0.0416	0.999	0.009
TFC (mg QE·g^−1^)	1.5984	−0.2166	1.1176	0.5239	−0.0869	−0.1266	0.4921	0.956	0.817
DPPH IC_50_ (mg·g^−1^)	7.3865	−0.5793	1.0946	0.5123	−0.2146	0.5155	−1.0341	0.926	1.424

$\beta_{0}$ is the intercept, $\beta_{1}$ is the linear coefficient of $x_{1}$ (sample–dispersant (*w*/*w*)), $\beta_{2}$ is the linear coefficient of $x_{2}$ (ethanol–water ratio (*v*/*v*)), $\beta_{3}$ is the linear coefficient of $x_{3}$ (vortex stirring time), $\beta_{12}$ is the interaction factor of $x_{1}$ and $x_{2}$, $\beta_{13}$ is the interaction factor of $x_{1}$ and $x_{3},$ and $\beta_{23}$ is the interaction factor of $x_{2}$ and $x_{3}$. Coefficient factors in bold are those with a *p*-value < 0.05, at the 95% confidence level, and which, therefore, have a significant effect on the study response. Data of TPC, TFC, and IC_50_ are expressed on a dry basis. IC_50_: concentration needed to reduce 50% of DPPH, GAE: gallic acid equivalent, QE: quercetin equivalent, and SEE: standard errors of estimation.

**Table 4:** Optimal values of the experimental extraction factors as a function of the response variables deduced by response surface analysis (RSA) and multiple response analysis (MRA).

Experimental Responses	Experimental Factors		
	Sample–Dispersant Ratio (*w*/*w*)	Ethanol–Water Ratio (*v*/*v*)	Vortex Stirring Time (min)
**RSA**			
TPC (mg GAE·g^−1^)	1/1	80:20	10
TFC (mg QE·g^−1^)	1/1	80:20	10
DPPH IC_50_ (mg·g^−1^)	1/5	20:80	5
**MRA**	1/1	80:20	10

RSA: response surface analysis, GAE: gallic acid equivalent, QE: quercetin equivalent, IC_50_: concentration needed to reduce 50% of DPPH, and MRA: multiple response analysis.

**Table 5:** Responses obtained under the optimal MSPD extraction conditions of cherry seeds before fermentation.

Experimental Responses	Experimental Factors			Responses		Comparison
	Sample–Dispersant Ratio (*w*/*w*)	Ethanol–Water Ratio (*v*/*v*)	Vortex Stirring Time (min)	Predicted	Experimental (Mean ± SD)	*t*_calc_
TPC (mg GAE·g^−1^)	1/1	80:20	10	1.268	1.37 ± 0.08	2.08
TFC (mg QE·g^−1^)				4.162	3 ± 1	2.44
DPPH IC_50_ (mg·g^−1^)				8.237	7.4 ± 0.3	5.22

The statistical analysis of the two-tailed Student’s *t*-test at a confidence level of 95% was used for the comparison of the values predicted by the model with the experimental values (*t*_tab_ = 4.30 in all experimental responses). Experimental results are expressed as mean ± standard deviation (SD) (*n* = 3) on a dry basis.

**Table 6:** Identification of phenolic compounds by HPLC-ESI-QTOF-MS in defatted cherry seed extracts.

Peak Number	Compound	RT (min)	Molecular Formula	[M-H]^−^ (*m*/*z*)	MS/MS Fragments (*m*/*z*)
1	Gallic acid ^a^	2.7	C_7_H_6_O_5_	169.1128	**125.0306**; 97.0302; 79.0243; 51.0305
2	2,3-Dihydroxybenzoic acid ^a^	4.9	C_7_H_6_O_4_	153.1138	125.5103; **109.0303**; 91.0201; 65.006
3	Neochlorogenic acid ^b^	4.9	C_16_H_18_O_9_	353.0879	**191.0433**; 179.0335; 161.1005; 135.0433; 107.0429
4	Chlorogenic acid ^a^	7.8	C_16_H_18_O_9_	353.3015	319.6037; 257.5812, **191.0613**; 161.0299; 85.0372
5	Vanillic acid ^a^	9.2	C_8_H_8_O_4_	167.0351	151.0007; **108.0227**; 91.0128; 80.5217
6	Caffeic acid ^a^	9.6	C_9_H_8_O_4_	179.1502	135.0469; 117.0299, 89.0406; 65.0374
7	Syringic acid ^a^	9.7	C_9_H_10_O_5_	197,0456	181.0194; 167.0176; 151.9915; 138.0294; **123.0072**; 95.0121
8	*p*-Coumaric acid ^a^	13.8	C_9_H_8_O_3_	163.1508	**119.0516**; 93.0364; 65.0359
9	*trans*-Ferulic acid ^a^	15.2	C_10_H_10_O_4_	193.1768	150.0655; **134.0400**; 106.0488; 89.0389
10	Kaempferol 3-rutinoside ^b^	17.7	C_27_H_30_O_15_	593.1513	423.0438; 367.7453; **285.0372**; 195.1208; 61.9900
11	Isorhamnetin 3-rutinoside ^c^	17.9	C_28_H_32_O_16_	623.1618	577.2840; **315.0504**; 300.0258; 271.6939; 243.0098
12	Quercetin ^a^	27.0	C_15_H_10_O_7_	301.2285	273.0425; 229.0516; 179.0033; **151.0081**; 121.0317
13	Kaempferol ^a^	32.6	C_15_H_10_O_6_	285.2291	255.0218; 229.0400; 185.0529; 151.0335; **93.0308**

Bold fragments are the main ones. Compared with MS data obtained from standard ^a^, FooDB database ^b^, and MassBank database ^c^.

**Table 7:** Quantification of phenolic compounds by HPLC-ESI-QTOF-MS in defatted cherry seed extracts.

Compound	Initial Seed (µg·g^−1^)	Seed after Fermentation (µg·g^−1^)	Seed after Both Fermentation and Distillation (µg·g^−1^)
2,3-Dihydroxybenzoic acid ^A^	38 ± 3 ^a^	64 ± 8 ^b^	84 ± 9 ^b^
Neochlorogenic acid ^B^	36 ± 1 ^a^	12 ± 4 ^b^	3.9 ± 0.4 ^c^
Chlorogenic acid ^B^	2.2 ± 0.4 ^a^	7.7 ± 0.5 ^b^	1.7 ± 0.8 ^a^
Vanillic acid ^A^	*n.q.*	1.2 ± 0.3 ^a^	1.7 ± 0.2 ^a^
Caffeic acid ^B^	25.4 ± 0.4 ^a^	13.5 ± 0.5 ^b^	3 ± 1 ^c^
Syringic acid ^A^	*n.d.*	*n.d.*	*n.q.*
*p*-Coumaric acid ^C^	*n.d.*	2.7 ± 0.4 ^a^	1.8 ± 0.1 ^b^
*trans*-Ferulic acid ^D^	*n.q.*	*n.d.*	*n.d.*
Kaempferol 3-rutinoside ^E^	0.27 ± 0.03 ^a^	*n.d.*	*n.d.*
Isorhamnetin 3-rutinoside ^F^	0.48 ± 0.03 ^a^	0.015 ± 0.001 ^b^	0.035 ± 0.005 ^b^
Quercetin ^F^	16 ± 2 ^a^	8 ± 2 ^b^	5.8 ± 0.1 ^b^
Kaempferol ^E^	2.1 ± 0.1 ^a^	0.95 ± 0.02 ^b^	2.2 ± 0.1 ^a^
**Total phenolic acids**	78 ± 4 ^a^	92 ± 3 ^a^	96 ± 9 ^a^
**Total flavonoids**	16 ± 2 ^a^	8 ± 2 ^b^	5.8 ± 0.1 ^b^
**Total phenolics**	96 ± 7 ^a^	100 ± 4 ^a^	104 ± 9 ^a^

Standard calibration curves: A—2,3-dyhydroxibenzoic acid (y = (1.7 ± 0.1)·10^7^x + (1.0 ± 0.1)·10^6^, R^2^ = 0.9962, LOD = 1.1 μg·L^−1^ and LOQ = 3.7 μg·L^−1^); B—caffeic acid (y = (2.98 ± 0.07)·10^7^ x + (8.0 ± 0.7)·10^5^, R^2^ = 0.9990, LOD = 2.5 μg·L^−1^ and LOQ = 8.3 μg·L^−1^); C—*p*-coumaric acid (y = (1.7 ± 0.1)·10^7^x + (8.0 ± 0.7)·10^5^, R^2^ = 0.9964, LOD = 1.1 μg·L^−1^ and LOQ = 3.7 μg·L^−1^); D—*trans*-ferulic acid (y = (5.4 ± 0.1)·10^6^x + (1.0 ± 0.1)·10^5^, R^2^ = 0.9993, LOD = 1.7 μg·L^−1^ and LOQ = 5.7 μg·L^−1^); E—kaempferol (y = (3.6 ± 0.1)·10^7^ x + (1.0 ± 0.1)·10^6^, R^2^ = 0.9962, LOD = 1.4 μg·L^−1^ and LOQ = 4.7 μg·L^−1^); F—quercetin (y = (3.6 ± 0.1)·10^7^ x + (3 ± 1)·10^6^, R^2^ = 0.9965, LOD = 1.4 μg·L^−1^, and LOQ = 4.7 μg·L^−1^); *n.d.* = not detected; *n.q.* = not quantified. For each seed, mean values with different superscripts for the same analyte reveal significant differences at *p*-values < 0.05, according to ANOVA and Fisher LSD test.

## Data Availability

The data presented in this study are available on request from the corresponding author. The data are not publicly available due to privacy concerns.
